# A new, potential and safe neoadjuvant therapy strategy in epidermal growth factor receptor mutation-positive resectable non-small-cell lung cancer-targeted therapy: a retrospective study

**DOI:** 10.3389/fonc.2024.1349172

**Published:** 2024-02-13

**Authors:** Baoxing Liu, Xingyu Liu, Huifang Xing, Haibo Ma, Zhenyu Lv, Yan Zheng, Wenqun Xing

**Affiliations:** ^1^ Department of Thoracic Surgery, The Affiliated Cancer Hospital of Zhengzhou University, Henan Cancer Hospital, Zhengzhou, China; ^2^ Department of Geriatric Medicine, The First Affiliated Hospital of Zhengzhou University, Zhengzhou, Henan, China

**Keywords:** osimertinib, epidermal growth factor receptor, neoadjuvant targeted therapy, non-small cell lung cancer, resectable

## Abstract

**Background:**

Studies of epidermal growth factor receptor (EGFR) tyrosine kinase inhibitors (TKIs) in resectable non-small-cell lung cancer (NSCLC) have been conducted. The purpose of our study was to evaluate the benefits of osimertinib as neoadjuvant therapy for resectable EGFR-mutated NSCLC.

**Method:**

This retrospective study evaluated patients with EGFR mutations in exon 19 or 21 who received targeted therapy with osimertinib (80 mg per day) before surgery between January 2019 and October 2023 in Henan Cancer Hospital.

**Results:**

Twenty patients were evaluated, all of whom underwent surgery. The rate of R0 resection was 100% (20/20). The objective response rate was 80% (16/20), and the disease control rate was 95% (19/20). Postoperative pathological analysis showed a 25% (5/20) major pathological response rate and 15% (3/20) pathological complete response rate. In total, 25% (5/20) developed adverse events (AEs), and the rate of grades 3–4 AEs was 10% (2/20). One patient experienced a grade 3 skin rash, and 1 patient experienced grade 3 diarrhea.

**Conclusion:**

Osimertinib as neoadjuvant therapy for resectable EGFR-mutated NSCLC is safe and well tolerated. Osimertinib has the potential to improve the radical resection rate and prognosis.

## 1 Introduction

Lung cancer, with non-small-cell lung cancer (NSCLC) accounting for approximately 85% of all lung cancers, is one of the most common malignant tumors and the leading cause of cancer-related deaths worldwide (1). Surgery is an important treatment strategy for patients with early-stage resectable NSCLC. However, patients with central lung tumors, giant tumors, and mediastinal or hilar lymph node metastases may be ineligible for upfront surgery. Neoadjuvant therapy can kill circulating tumor cells in the blood, reduce tumor load, downgrade tumor stage, and allow the possibility of surgery, thereby improving long-term survival (2). A systematic meta-analysis of 15 trials on neoadjuvant chemotherapy in NSCLC showed that preoperative neoadjuvant chemotherapy could better reduce the relative mortality risk, reduce postoperative recurrence, and improve the 5-year survival rate (3).

Imaging tests can be used to evaluate the objective response rate (ORR) and the percentage of patients who achieve complete or partial response after treatment. Major pathological response (MPR) indicates less than 10% of residual tumor cells in the tumor tissue after resection, while pathological complete response (pCR) indicates that there are no cancer cells in the tumor tissue and lymph nodes after resection. Compared with the ORR, the pathological response rate is more important for the efficacy evaluation of neoadjuvant therapy and can better reflect the real situation after treatment (4). In a previous study, the MPR and pCR of neoadjuvant chemotherapy or radiotherapy was less than 10%, indicating limited efficacy (5, 6). In recent years, targeted therapy has become a first-line treatment strategy for advanced lung cancer. The potential of targeted therapy for neoadjuvant therapy in resectable NSCLC has also been investigated.

Epidermal growth factor receptor (EGFR) tyrosine kinase inhibitors (TKIs) can specifically identify tumor cells with EGFR mutations and block the signal transduction pathway of tyrosine protein kinase to promote tumor cell apoptosis (7). Compared with chemoradiotherapy, targeted therapy is more precise, has fewer side effects, and can provide significantly longer progression-free survival (PFS) (8). EGFR mutation, the most common target in patients with NSCLC, exists in 40%–60% of Asian patients and is common in women, nonsmokers, and patients with adenocarcinoma (9). In patients with EGFR-mutated NSCLC, in-frame deletion in exon 19 and amino acid substitution in exon 21 are present in approximately 90% (10, 11). EGFR-TKIs have become the first-line treatment strategy for patients with advanced NSCLC owing to their survival benefits in large-scale randomized controlled trials.

Many recent studies have explored the potential of EGFR-TKI as a neoadjuvant therapy for resectable NSCLC. The phase II CTONG 1103 trial showed better PFS benefits of neoadjuvant erlotinib than chemotherapy (12). The phase II NCT01217619 study also indicated that erlotinib had better survival benefits (13). In the Ascent study, afatinib obtained better MPR and pCR rate, as well as better PFS and OS time (14). The NEOS study updated the results of osimertinib as neoadjuvant therapy in patients with EGFR-mutated resectable stage II-IIIB lung adenocarcinoma at the 2022 European Society for Medical Oncology (ESMO) Congress, thus, further supporting the advantages of osimertinib over other EGFR-TKIs (15).

EGFR-TKIs have presented the situation of “three generations under one roof.” The FLAURA study showed that for PFS, osimertinib, the representative third-generation EGFR-TKI, is superior to the first-generation EGFR-TKIs gefitinib and erlotinib (16). Moreover, osimertinib is the first EGFR-TKI to obtain a median OS benefit of 38.6 months (17). Osimertinib also has a significant therapeutic advantage in cancer patients with the central nervous system involvement (18). As a kind of small mono-anilino pyrimidine molecule, osimertinib is an oral, irreversible, selective inhibitor targeting EGFR activation and resistance (T790M) mutations. The acrylamido of osimertinib can bond covalently with C797 amino acids on the edge of the ATP binding site of the EGFR gene catalytic domain, becoming irreversibly bound to specific EGFR mutation forms (L858R, 19Del and double mutation containing T790M). The irreversible bond inhibits EGFR kinase phosphorylation and activation of downstream tumor signaling pathways and induces EGFR-mutated cells to degrade. Currently, osimertinib is the standard first-line TKI for advanced EGFR-mutated NSCLC. However, despite these numerous benefits, it remains unclear whether neoadjuvant EGFR-TKIs could better improve OS in comparison to chemotherapy owing to the sample sizes in previous studies and the lack of large phase III clinical trials. Thus, in this study, we aimed to evaluate the efficacy and safety of the neoadjuvant osimertinib in patients with resectable NSCLC to provide a basis for its use in the neoadjuvant setting.

## 2 Methods

### 2.1 Study design and patients

This was a single-arm retrospective study of osimertinib as a neoadjuvant therapy in patients with NSCLC with EGFR 19 exon or 21 exon mutations. Patients with NSCLC who received neoadjuvant osimertinib targeted therapy followed by surgical resection at the Affiliated Cancer Hospital of Zhengzhou University (Henan Cancer Hospital) between January 2019 and October 2023 were evaluated. The inclusion criteria were as follows: (1) an established diagnosis of NSCLC; (2) detection of EGFR mutations and EGFR exon 19 or 21 mutations; (3) neoadjuvant therapy with osimertinib followed by surgery; and (4) no history of other malignancies.

In this study, patients would undergo a series of pre-operative imaging tests to assess the tumor staging, which include chest CT, brain MRI, ECT, abdominal and neck ultrasonography. What’s more, PET-CT might be an additional imaging test for a small percentage of patients due to its’ expensiveness and self-paying. Then patients received different durations of neoadjuvant osimertinib therapy (80 mg/day) followed by surgical resection. All patients refused chemotherapy. Radiographic and pathologic responses after osimertinib induction treatment were jointly assessed by thoracic surgeons, radiologists, and pathologists according to the Response Evaluation Criteria in Solid Tumors.

This study was approved by the research ethics committee of the Affiliated Cancer Hospital of Zhengzhou University (Henan Cancer Hospital) and was conducted according to the tenets of the Helsinki Declaration.

### 2.2 Statistical analysis

We performed a descriptive statistical analysis, chi-square test, Fisher’s exact test, univariate and multivariate analyses. All statistical analyses were performed using SPSS (version 25.0; IBM Corp., Armonk, NY, USA). Relevant charts were generated using GraphPad Prism 7.0.

## 3 Results

### 3.1 Patient characteristics

In total, twenty patients (12 males and 8 females) were evaluated. The mean patient age was 56.2 ± 10.0 years, and eight and twelve patients were aged 30–55 years and 55–80 years, respectively. Five patients had a history of smoking, and five and three patients had Eastern Cooperative Oncology Group Performance Status scores of 1 and 2, respectively. In eight patients, tumors were located in the hilar region, and clinical lymph node metastasis was found in fifteen patients preoperatively. The tumor pathology was lung adenocarcinoma in eighteen patients; lung squamous cell carcinoma in one patient; and adenocarcinoma mixed with neuroendocrine carcinoma, which was postoperatively diagnosed as large cell neuroendocrine carcinoma, in another patient. Fourteen patients were preoperatively diagnosed using percutaneous lung puncture biopsy, and the other six patients were diagnosed preoperatively using bronchoscopy. The mean duration of neoadjuvant osimertinib was 58.3 ± 18.7 days. Eighteen patients underwent lobectomy, and two patients underwent pneumonectomy. The mean surgery time was 177 ± 53 min. Postoperative pathology showed invasion of the pleura in 9 patients, lymph node metastasis in 11 patients, pathological changes in cell degeneration in 11 patients, and pathologic complete response in 3 patient. Postoperative chylothorax occurred in 1 patient. The baseline demographic and clinical characteristics are shown in **Tables 1**, **2**, respectively.

**Table 1:** Baseline patient demographics and clinical characteristics.

Characteristics			Characteristics		
Gender			Tumor(T)		
	Male	12		T1	11
	Female	8		T2	9
Mean age		56.2 ± 10.0	LN metastasis(N)		
	30-55	8		N0	9
	55-80	12		N1	3
Smoking Status				N2	8
	Yes	5	Metastasis(M)		
	No	15		M0	20
Performance Status				M1	0
	ECOG=0	12	Invasion of pleura		
	ECOG=1	5		Yes	9
	ECOG=2	3		No	11
Pathology			Surgery time(min)		177 ± 53
	LUAD	18		60-180	12
	LUSC	1		180-300	8
	LCNEC	1	Therapy duration(day)		58.3 ± 18.7
Pathological methods				30-55	12
	Percutaneous pulmonary biops	14		56-120	8
	Bronchoscopy	6	ORR		80%
EGFR Mutation status				PR	15
	exon 19 del	11		CR	0
	exon 21 L858R	9	SD		3
AEs			PD		1
	grade1-2	3	DCR		95%
	grade3-4	2	Pathology response		
Surgical method				MPR	5
	Lobectomy	18		PCR	3
	Pneumonectomy	2	R0 resection		20

LUAD, lung adenocarcinoma; LUSC, lung squamous cell cancer; LCNEC, lung neuroendocrine cancer; AEs, adverse events; LN, lymphonodus; ORR, objective response rate; PR, partial response; SD, stable disease; CR, complete response; PD, progressive disease; MPR, major pathologic response; PCR, pathologic complete response; ±, standard deviation; del, deletion.

**Table 2:** Clinical data of 20 patients receiving osimertinib neoadjuvant targeted therapy.

No.	TNM Stage*	Exon	ECOG	Therapy duration	Tumor size reduction (%)	Surgical options	LN Metastasis	Response	Bleeding(ml)
1	T2bN0M0, IIa	19	2	60	51.4	Lobectomy	No	PCR	200
2	T2bN0M0, IIa	19	1	40	54.5	Lobectomy	No	PR	100
3	T2N0M0, Ib	21	1	55	52.1	Lobectomy	No	PR	1100
4	T2N1M0, IIb	19	1	35	54.5	Pneumonectomy	Yes	PR	100
5	T2aN0M0, Ib	21	1	35	23.5	Lobectomy	No	PR	100
6	T2N2M0, IIIa	12	2	80	78.3	Lobectomy	Yes	–	150
7	T2N1M0, IIb	21	2	35	-46.2	Pneumonectomy	Yes	MPR	40
8	T2aN2M0, IIIa	19	1	50	40.0	Lobectomy	Yes	PR	80
9	T1cN0M0, Ia	19	0	55	9.5	Lobectomy	No	PR	500
10	T1cN2M0, IIIa	19	0	80	30.8	Lobectomy	Yes	PR	60
11	T1bN2M0, IIIa	21	0	60	28.6	Lobectomy	Yes	–	100
12	T1cN0M0, Ia	19	0	55	58.6	Lobectomy	No	PCR	100
13	T1cN0M0, Ia	19	0	55	38.5	Lobectomy	No	–	100
14	T2aN1M0, IIb	21	0	50	25.0	Lobectomy	Yes	–	100
15	T1cN2M0, IIIa	21	0	50	33.3	Lobectomy	Yes	–	200
16	T1cN2M0, IIIa	21	0	50	44.4	Lobectomy	Yes	–	100
17	T1bN0M0, Ia	19	0	110	73.7	Lobectomy	No	PR	100
18	T1bN2M0, IIIa	19	0	60	68.2	Lobectomy	Yes	MPR	100
19	T1bN2M0, IIIa	19	0	65	47.6	Lobectomy	No	PR	150
20	T1aN0M0, Ia	21	0	85	57.1	Lobectomy	No	PCR	100

*pathological TNM staging; PCR, pathologic complete response; PR, pathologic response≧50%; MPR, major pathologic response; SD, stable disease.

### 3.2 Efficacy

**Figure 1:**
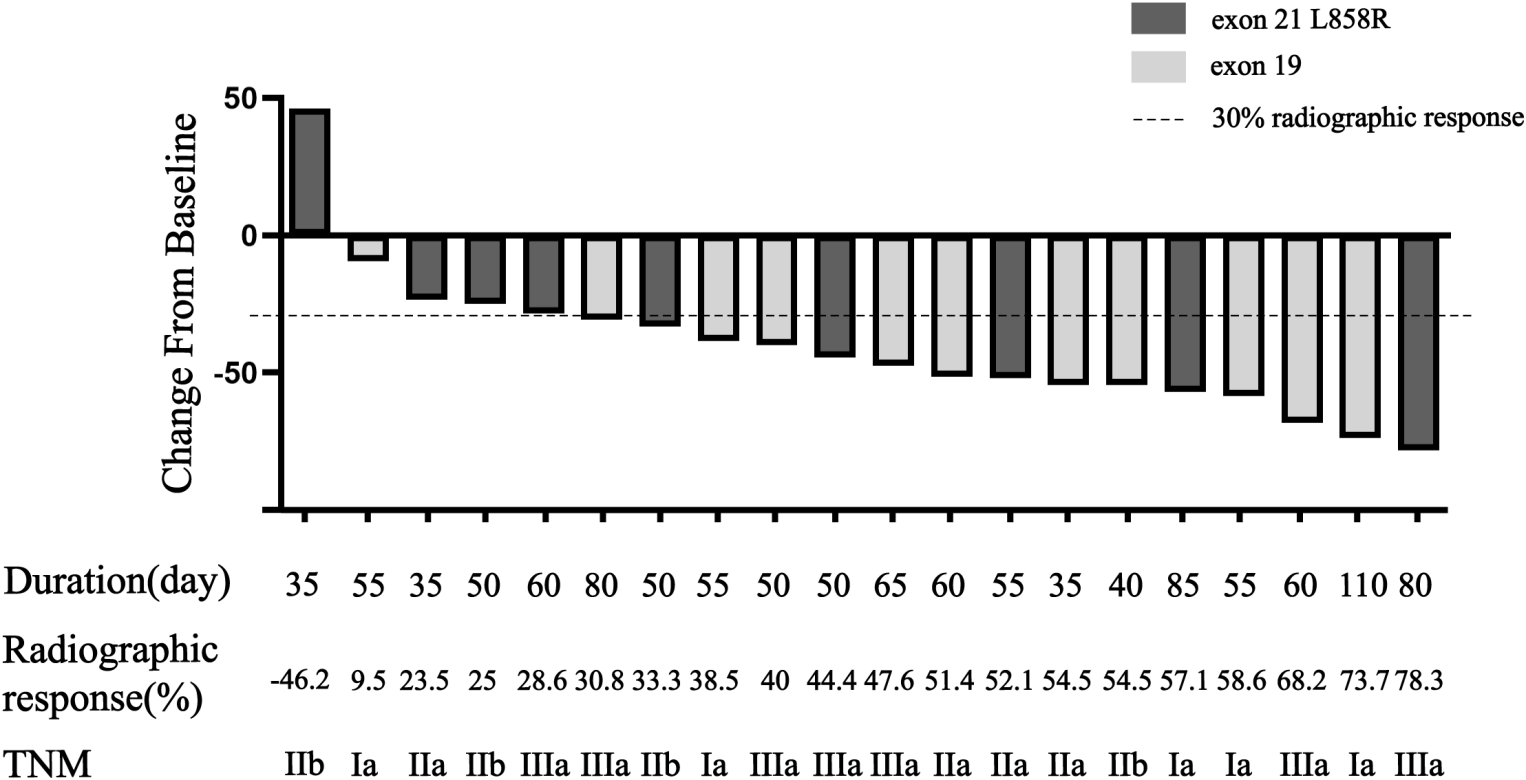
Waterfall plot of response to osimertinib neoadjuvant therapy. Bars show data from individual patients. Negative values suggest tumor shrinkage and positive values suggest PD.

**Figure 2:**
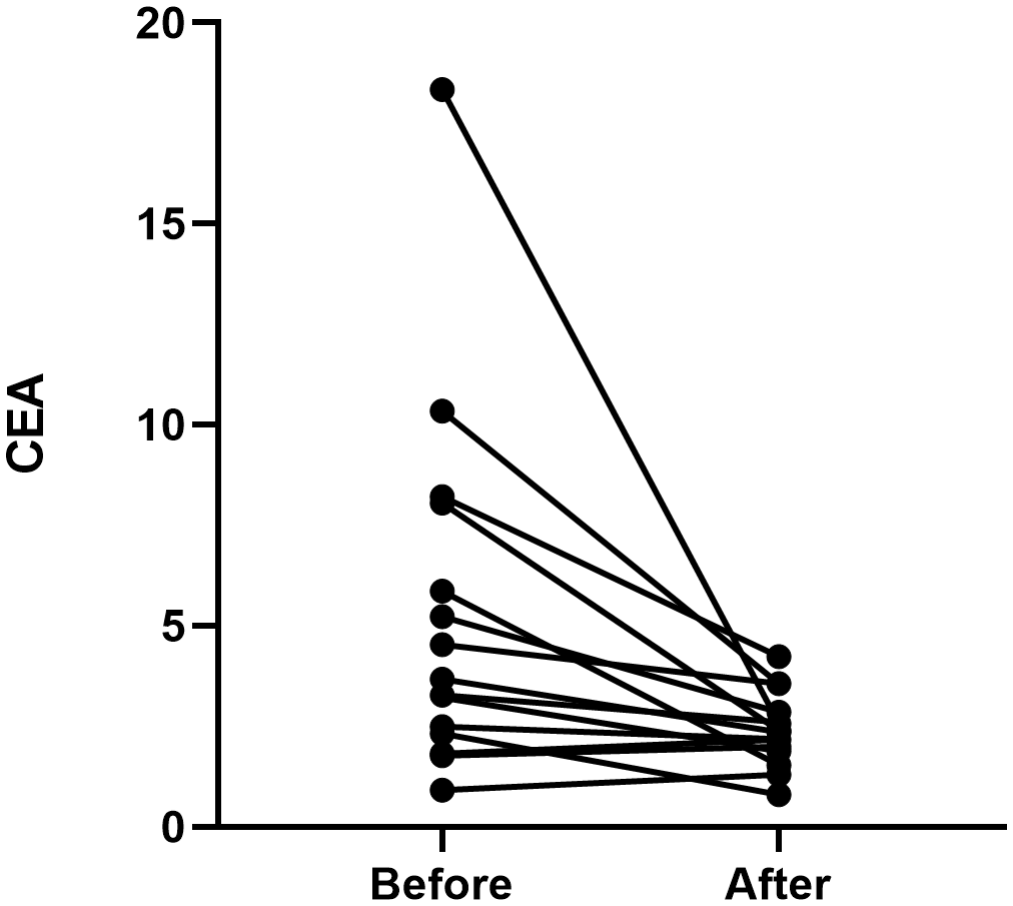
CEA level before and after osimertinib neoadjuvant therapy for eight cases. CEA, carcinoembryonic antigen.

**Figure 3:**
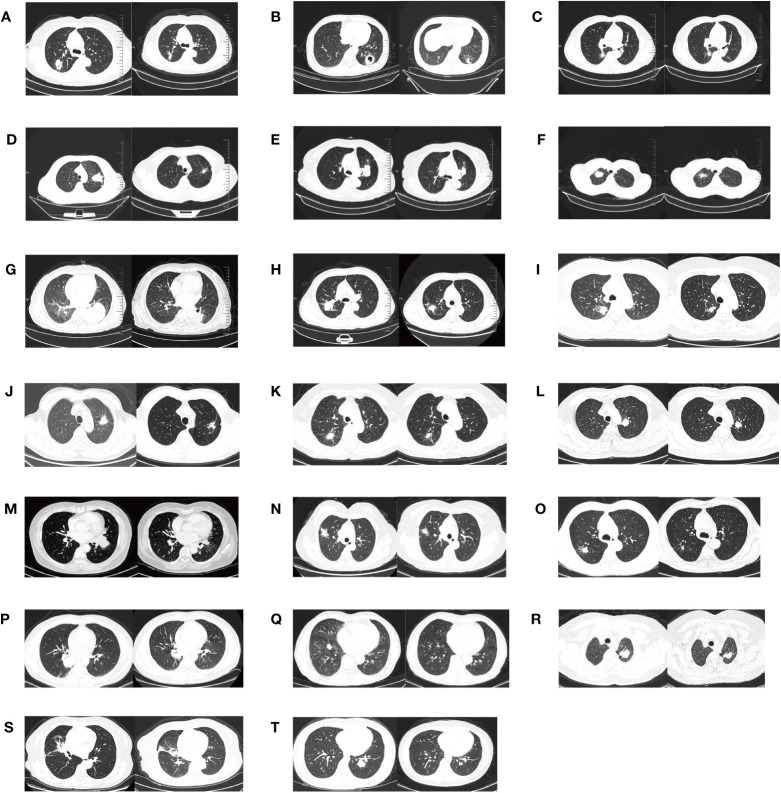
The radiologic evaluation before and after osimertinib neoadjuvant therapy for twenty cases. **(A–T)** means the serial number of twenty patients.

**Figure 4:**
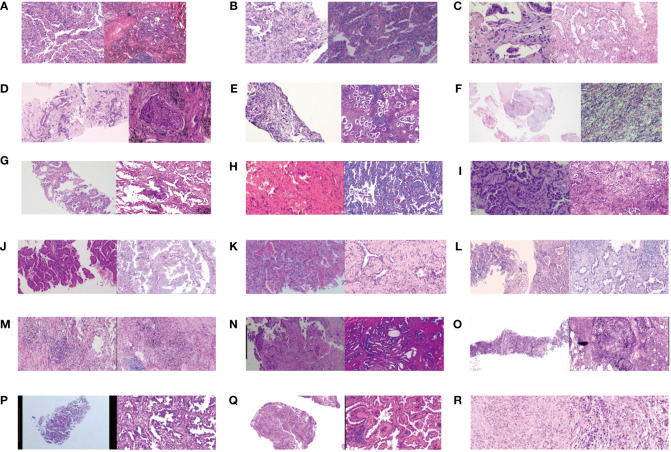
The pathologic evaluation before and after osimertinib neoadjuvant therapy for eighteen cases. **(A–R)** means the serial number of eighteen patients. Clear pathological images were missing in two of twenty patients.

All twenty patients who underwent neoadjuvant osimertinib therapy ultimately underwent surgery, and the rate of R0 resection was 100% (20/20). Meanwhile, the disease control rate in the current study was 95% (19/20). The tumor size decreased and increased in nineteen and one patient, respectively, but the hilar lymph nodes of this one patient decreased. Treatment response to neoadjuvant osimertinib is shown as a waterfall plot in **Figure 1**. We obtained an ORR of 75% (15/20). The levels of tumor markers, such as carcinoembryonic antigen and cytokeratin-19-fragment, were reduced in fifteen patients. The other patients did not undergo a blood test for tumor markers before or after targeted therapy (**Figure 2**). Pathological examination and immunohistochemistry of postoperative tumor tissues showed that three patient achieved pCR, while eleven patients had tumor cell regression. The MPR rate was 25% (5/20), and the pCR rate was 15% (3/20). The results of this study are compared with those of previous studies in **Table 3**. The radiologic and pathologic evaluation before and after osimertinib neoadjuvant therapy are shown in **Figures 3**, **4**.

**Table 3:** The contrast information between the retrospective study and other studies.

Study	The study	Zhong	P	Xiong	P	Zhang	P	Rizvi	P	Zhong	P
Enrollment years	2019-2022	2011-2017		2011-2014		2013-2015		2004-2008		2008-2011	
Case number	8	37		19		35		21		12	
Clinical stage	IIA-IIIA	IIIA		IIIA		II-IIIA		IA-IIB		IIIA	
Target drug	Osimertinib	Erlotinib		Erlotinib		Gefitinib		Gefitinib		Erlotinib	
Duration (day)	48.6 ± 16.2	42		56		42		21		42	
R0 resection	100%(20/20)	73.0%(27/37)	0.010	68.4%(13/19)	0.008	82.9%(29/35)	0.130	nr		25%(3/12)	<0.001
ORR	75%(15/20)	54.1%(20/37)	0.084	42.1%(8/19)	0.022	54.5%(19/35)	0.082	42%(21/50)	0.007	58.3%(7/12)	0.240
AEs	25%(5/20)	75.7%(28/37)	<0.001	36.8%(7/19)	0.501	85.7%(30/35)	<0.001	nr		100%(12/12)	<0.001
AEs (grade 3-4)	10%(2/20)	0	0.229	15.8%(3/19)	0.661	0	0.247	nr		16.7%(2/12)	0.620
MPR	25%(5/20)	nr		nr		12.1%(4/33)	0.272	nr		nr	
PCR	15%(3/20)	9.7%(3/31)	0.896	nr		24.2%(8/33)	0.503	nr		nr	

ORR, objective response rate; AEs, adverse events; MPR, major pathologic response; PCR, pathologic complete response; nr, not reached.

### 3.3 Safety and tolerance

Although traditional chemotherapy has good efficacy in neoadjuvant therapy, it is accompanied by serious adverse events (AEs). Therefore, it is important to find an effective drug with fewer side effects. This retrospective study showed that osimertinib is safe and tolerable in neoadjuvant therapy. Five patients developed grade AE throughout the course of neoadjuvant osimertinib therapy. The grade 1/2 AEs include two cases of oral ulcer, and one skin rash. The rate of grade 3 AEs was 10% (2/20), one grade 3 diarrhea and one grade 3 rash. No patient experienced grade ≥4 AEs, and no patient died from treatment-related AEs.

### 3.4 Subgroup analysis

Subgroup analyses were performed according to age, sex, gene mutations, and therapy duration (**Table 4**). Compared with men, women showed lower ORR rate (75% vs. 87.5%, p=0.47) but higher pCR rate (16.7% vs. 12.5%, p=0.62). This indicated that female patients might benefit more from neoadjuvant osimertinib therapy, although the difference is not significant. There were twelve patients aged >55 years and eight patients aged <55 years. The ORR of the eight younger and the twelve older patients were 62.5% and 83.3% (p=0.35); and pCR rate, 12.5% and 16.7% (p=0.62), respectively, with no significant difference. Eleven patients had EGFR exon 19 mutation and the other nine patients had EGFR exon 21 L858R mutation. The ORR were 91% and 55.6% (p=0.13); and pCR, 18% and 11% (p=0.58), respectively. There was no significant difference of ORR and pCR benefits between patients with EGFR exon 19 mutation and EGFR exon 21 L858R mutation. The treatment duration was <55 days and >55 days in twelve and eight patients, respectively. The ORR for those with shorter and longer therapy durations were 66.7% and 87.5% (p=0.60); and pCR rate, 8.3% and 25% (p=0.34), respectively. This indicated that a longer duration of neoadjuvant osimertinib therapy was more beneficial.

**Table 4:** Subgroup analyses.

Subgroup				P
Gender		Male	Female	
	ORR	87.5	75	0.47
	PCR	12.5	16.7	0.62
Age		<55	>55	P
	ORR	62.5	83.3	0.35
	PCR	12.5	16.7	0.62
EGFR mutation		exon 19	exon 21	P
	ORR	91	55.6	0.13
	PCR	18	11	0.58
Therapy duration		30-55	56-120	P
	ORR	66.7	87.5	0.60
	PCR	8.3	25	0.34

ORR, objective response rate; PR, partial response; PCR, pathologic complete response.

## 4 Discussion

Surgical resection is the preferred treatment modality for patients with early stage and locally advanced NSCLC. Previous studies have indicated that the 5-year survival rate of patients with stage III NSCLC is less than 30%, while those with stage IB disease is 70% (19). Frei et al. (20) first proposed neoadjuvant chemotherapy for patients with solid tumors. Neoadjuvant therapy can reduce the tumor burden to achieve preoperative tumor reduction, providing patients the opportunity to receive R0 resection and optimize the surgical benefit. Neoadjuvant therapy can also effectively remove existing micro-metastatic lesions, reduce the probability of recurrence, and prolong survival. The update data of ADAURA study show that in stage II–IIIA patients, osimertinib could bring longer DFS (65.8 months vs 21.9 months; HR, 0.23; 95% CI, 0.18-0.30), and the risk of CNS metastases was reduced by 76%. Adjuvant osimertinib showed an overwhelmingly positive effect on DFS benefit and reducing CNS metastases risk, further supporting osimertinib as a better adjuvant therapy and a new therapy standard for patients with EGFR-mutated NSCLC. Compared with chemotherapy, targeted therapy has higher response rates and fewer AEs and is thus recommended as a first-line treatment modality for advanced NSCLC. Accordingly, there have been several studies on the benefits and risks of targeted therapy as neoadjuvant therapy. The present study demonstrates that for potentially resectable NSCLC with EGFR mutations, neoadjuvant EGFR-TKI therapy with osimertinib is a safe and feasible treatment strategy, as evidenced by a satisfactory R0 resection rate and low toxicity. The findings need to be confirmed in further phase III clinical trials owing to the limited sample size.

### 4.1 Effectiveness of neoadjuvant EGFR-TKI for EGFR-mutant NSCLC

The present study shows that all patients who received neoadjuvant osimertinib achieved R0 resection, and 2 cases of grades 3–4 AEs were observed. In the phase II CTONG 1103 (EMERGING) trial (12), among the 72 patients with stage IIIA-N2 NSCLC, those who received neoadjuvant erlotinib showed better PFS than did patients who received chemotherapy (gemcitabine plus cisplatin) (21.5 months vs 11.4 months, P<0.001). In the single-arm phase II NCT01217619 clinical trial, 19 stage IIIA-N2 patients with EGFR mutations received neoadjuvant erlotinib. The R0 resection rate was 68.4% (13/19), the ORR was 42.1%, and the rate of pathological downstaging after surgery was 21.1%. The median PFS was 11.2 months, and the median OS was 51.6 months (13). In the phase II Ascent study (14), which recruited patients with stage III EGFR-mutated NSCLC, the MPR rate was 57.1% (4/7), and the pCR rate was 14.3% (1/7) in 7 patients who underwent surgery. The ORR of 13 participants was 69%, the median PFS was 34.6 months, and the 2-year OS rate was 85%.

The NEOS study updated the results of osimertinib as neoadjuvant therapy in patients with EGFR-mutated resectable stage II–IIIB lung adenocarcinoma in the 2022 European Lung Cancer Congress (16). In 38 patients who received 6-week neoadjuvant osimertinib, the ORR was 71.1% (27/38), and the disease control rate was 100%. In 28 patients with evaluable pathology, the MPR was 11% (3/28), and the pCR was 4% (1/28). Further, 46% (13/28) of patients showed a pathological response of ≥50%. In 32 patients who underwent surgery, the R0 resection rate was 94% (30/32). A meta-analysis of five trials (12, 21–24) compared erlotinib to chemotherapy as neoadjuvant therapy for patients with stage IIIA EGFR-mutated NSCLC. The results indicated that erlotinib was superior to chemotherapy in OS (hazard ratio, 0.74; 95% confidence interval [CI], 0.43–1.27) and PFS (hazard ratio, 081; 95% CI, 0.27–2.44) (25). There was also a trend toward favorable ORR (risk ratio, 1.70; 95% CI, 1.35–2.15), progression rate (risk ratio, 0.64; 95% CI, 0.34–1.19), and OpR (risk ratio, 1.13; 95% CI, 1.01–1.26), which resulted in significant improvements in patient prognosis.

Collectively, data from clinical trials indicated that neoadjuvant EGFR-TKIs, including erlotinib, afatinib, and osimertinib, could improve the pathological response rate and PFS of NSCLC patients. Compared to the first and second generation of EGFR-TKIs, osimertinib has better safety profile and compliance. The main thing is that osimertinib can bring longer PFS and OS benefits according to the FLAURA trial ans ADAURA trial. Therefore, osimertinib is worthwhile further study in neoadjuvant therapy. Larger randomized controlled trials are needed to further clarify the efficacy of osimertinib as a neoadjuvant therapy for NSCLC.

### 4.2 Safety of neoadjuvant EGFR-TKIs and their impact on surgery

Patients with EGFR mutations generally have good tolerability to neoadjuvant EGFR-TKI treatments. Common AEs include skin rashes, diarrhea, and hepatotoxicity. A meta-analysis of five clinical trials on neoadjuvant EGFR-TKIs indicated that patients with EGFR-mutated stage IIIA NSCLC were extremely sensitive to neoadjuvant EGFR-TKI, and the incidence of grade 3/4 AEs in the targeted therapy group was significantly lower than that in the neoadjuvant chemotherapy group (26). The major AE for patients who received neoadjuvant targeted therapy was skin rash, while those for patients receiving neoadjuvant chemotherapy were gastrointestinal symptoms and myelosuppression (27). Neoadjuvant targeted therapy in a preoperative setting is also safe. The potential application of neoadjuvant EGFR-TKIs for the increasing the feasibility of surgery has also been evaluated. In the NEOS study, the rate of R0 resection was 94% (30/32) (16). In the experimental group of the CTONG 1103 study, the rate of patients who underwent lobectomy was 64.9% and the rates of bilobectomy and pneumonectomy were 13.5% and 5.4%, respectively (12). In the study by Zhang et al., the rates of lobectomy and bilobectomy were 93.9% and 6.1%, respectively (23). Overall, surgery is feasible after neoadjuvant EGFR-TKI therapy.

Another concern is whether neoadjuvant EGFR-TKIs increases the difficulty of surgery. A previous study indicated that neoadjuvant chemotherapy increases the difficulty of tumor resection and dissection due to tissue edema, adhesions, and fusion of tissue planes (28). This needs to be clarified in neoadjuvant targeted therapy. Questions about whether neoadjuvant targeted therapy increased the difficulty of surgery and operation times were not addressed in the few clinical trials involving EGFR-TKI neoadjuvant treatment. Therefore, we could not compare our study results with those of previous clinical trials. Although our study provided some indices for evaluating the difficulty of surgery, such as operation time and bleeding volume, the findings could not be compared with previous studies owing to differences in cancer type, surgery and surgeons, and surgical protocol. However, operative time and blood loss, which reflect the degree of surgical difficulty, were not significantly different between two neoadjuvant treatment strategies in a previous study (29). Bott et al. indicated that the median operative times of the two neoadjuvant treatment strategies were similar (30). However, in the study by Jiang et al., only 9 of 32 patients underwent minimally invasive surgery (31). Further large clinical trials and additional clinical data are needed to assess the impact of neoadjuvant osimertinib therapy on operative time, surgical difficulty, and perioperative AEs.

Moreover, the postoperative mortality rate was 0%, and there were few postoperative AEs. Two studies reported a 3%–5% deterioration of pulmonary function after neoadjuvant erlotinib (32). A 7% postoperative chylothorax rate was also reported in a retrospective study (33), and one patient experienced chylothorax in our study. The high rate of postoperative chylothorax may have been a bias caused by the small study size. Surgery-related risks and difficulties, such as surgical delay and cancer progression, and perioperative AEs caused by neoadjuvant targeted therapy are of great concern to surgeons. Further accurate selection of suitable patients and exploration of a more efficient and less toxic combination therapy are needed for neoadjuvant therapy in NSCLC.

### 4.3 Therapy duration of neoadjuvant EGFR-TKI

Another concern is the duration of targeted treatment in the neoadjuvant setting for patients with EGFR-mutated NSCLC. In the current study, the average duration of neoadjuvant osimertinib before surgery was 58.3 ± 18.7 days (range, 35–110 days), and all 20 patients underwent R0 resection. In the CTONG 1103 study, 35 patients in the targeted therapy group received 42-day erlotinib, and the median PFS was 21.5 months, and the ORR was 54.1% (12). However, in the CSLC0702 study, in which patients also received erlotinib for 42 days, the median PFS was only 6.9 months (24). In a study by Xiong et al., where patients received erlotinib for 56 days, the median PFS was 11.2 months, and the ORR was 42% (21). In a meta-analysis of 5 clinical trials, the median duration of targeted therapy was 42 days (range, 21–56 days), and the ORR ranged from 42% to 81% (25). The authors suggested that 42 days might be a rational medication duration for neoadjuvant EGFR-TKIs based on the ORR and postoperative outcomes (25), and similar results were found in the current analysis. A therapy time-window of 40–50 days would allow an appropriate time for evaluation of efficacy and acceptable toxicities and ensure that surgical intervention is not inappropriately delayed (25). In addition to therapy duration, the withdrawal time is another advantage of neoadjuvant EGFR-TKIs over neoadjuvant chemotherapy. In general, neoadjuvant therapy is terminated 4–6 weeks before surgery. Neoadjuvant targeted therapy could shorten the withdrawal time to ≤2 weeks.

### 4.4 Monotherapy versus combination therapy

EGFR-TKIs have become the first-line treatment strategy for patients with advanced EGFR-mutated NSCLC. However, it remains unclear whether neoadjuvant targeted monotherapy or targeted therapy combined with chemotherapy is more beneficial for patients with locally advanced cancer. Chen et al. and Xiong et al. demonstrated that neoadjuvant EGFR-TKI monotherapy is superior to neoadjuvant chemotherapy with respect to ORR and OS (13, 28). A more effective neoadjuvant strategy for EGFR-TKI therapy is needed to explore and verify the less-than-optimal pCR for EGFR-TKIs. The NEJ009 study, the first phase III study to compared between EGFR TKI combined with chemotherapy and EGFR TKI monotherapy in advanced NSCLC, showed a significantly higher ORR (84% vs 67%), longer median PFS (20.9 months vs 11.9 months), and longer OS (50.9 months vs 38.8 months) in the combination group (34). A phase III study by Noronha et al. reported similar treatment benefits (35). These results indicate that EGFR-TKI combined with chemotherapy as a neoadjuvant strategy is worth exploring, and further clinical trials are needed to validate its feasibility. The ongoing NEOADAURA study which compared neoadjuvant osim +/- chemotherapy versus chemotherapy in patients with resectable EGFR positive NSCLC may bring the final answer.

Targeted therapy is not routinely recommended as perioperative neoadjuvant therapy in the treatment guidelines for resectable NSCLC. However, previous phase II clinical trials on neoadjuvant targeted therapy have shown initial success. For patients with EGFR mutations, especially in Asian populations, neoadjuvant targeted therapy has advantages over neoadjuvant chemotherapy with respect to efficacy and safety. With its benefits of low toxicity, high rate of downstaging, and high rate of tumor regression, targeted therapy might offer better options for neoadjuvant therapy. Neoadjuvant therapy should not be aimed at improving the resectability of surgically difficult tumors but should be aimed at improving OS and DFS by increasing pathologic regression and reducing distant relapse.

This study has some obvious limitations. Firstly, the retrospective study was a single-arm research study from a single cancer center. Secondly, the study is limited by a small sample size and little examination of outcomes. Multicenter and large studies are needed. Third, because this is a retrospective study and no systematic follow-up of patients was performed after surgery, there was no data on DFS or OS. And The association between pathological response and recurrence/survival has not been clearly established in EGFR mutation positive NSCLC.

In conclusion, neoadjuvant osimertinib targeted therapy appears to be the optimal treatment strategy for patients who do not want to receive chemotherapy owing to its low toxicity, high rate of downstaging, and high rate of pathologic regression. Further prospective clinical trials are necessary to verify the efficacy of neoadjuvant EGFR-TKI therapy and determine the optimal therapeutic TKI.

## Data availability statement

The authors confirm that the data supporting the findings of this study are available within the article and its Supplementary Materials.

## Ethics statement

The studies involving humans were approved by the research ethics committee of the Affiliated Cancer Hospital of Zhengzhou University. The studies were conducted in accordance with the local legislation and institutional requirements. Written informed consent for participation was not required from the participants or the participants’ legal guardians/next of kin in accordance with the national legislation and institutional requirements.

## Author contributions

BL: Writing – review & editing. XL: Writing – original draft. HX: Writing – review & editing. HM: Software, Writing – review & editing. ZL: Writing – original draft. YZ: Writing – review & editing. WX: Writing – review & editing.
